# Spiritual health practitioners’ contributions to psychedelic assisted therapy: A qualitative analysis

**DOI:** 10.1371/journal.pone.0296071

**Published:** 2024-01-02

**Authors:** Caroline Peacock, Jennifer S. Mascaro, Erin Brauer, Ali John Zarrabi, Boadie W. Dunlop, Jessica L. Maples-Keller, George H. Grant, Charles L. Raison, Fayzan Rab, Roman Palitsky

**Affiliations:** 1 Winship Cancer Institute, Emory University, Atlanta, Georgia, United States of America; 2 Department of Spiritual Health, Emory University Woodruff Health Sciences Center, Atlanta, Georgia, United States of America; 3 Emory Center for Psychedelics and Spirituality, Emory University, Atlanta, Georgia, United States of America; 4 Department of Family and Preventive Medicine, Emory University School of Medicine, Atlanta, Georgia, United States of America; 5 Division of Palliative Medicine, Department of Family and Preventive Medicine, Emory University School of Medicine, Atlanta, Georgia, United States of America; 6 Department of Psychiatry and Behavioral Sciences, Emory University School of Medicine, Atlanta, Georgia, United States of America; 7 Nell Hodgson Woodruff School of Nursing, Emory University, Atlanta, Georgia, United States of America; 8 Emory University School of Medicine, Atlanta, Georgia, United States of America; Pontificia Universidad Catolica de Chile (PUC) / Universidad de Valladolid (UVa), SPAIN

## Abstract

**Background:**

Psychedelic-assisted therapies hold early promise for treating multiple psychiatric conditions. However, absent standards for the care, teams providing psychedelic-assisted therapy pose a major roadblock to safe administration. Psychedelics often produce spiritually and existentially meaningful experiences, and spiritual health practitioners have been involved in administering psychedelic-assisted therapies in multiple settings, suggesting important qualifications for delivering these therapies. However, the roles and competencies of spiritual health practitioners in psychedelic-assisted therapies have not been described in research.

**Method:**

This study examined interviews with 15 spiritual health practitioners who have facilitated psychedelic-assisted therapy. Thematic analyses focused on their contributions, application of expertise and professional background, and roles in administering these therapies.

**Results:**

Seven themes emerged, comprising two domains: unique and general contributions. Unique contributions included: competency to work with spiritual material, awareness of power dynamics, familiarity with non-ordinary states of consciousness, holding space, and offer a counterbalance to biomedical perspectives. General contributions included use of generalizable therapeutic repertoire when conducting PAT, and contributing to interdisciplinary collaboration.

**Implications:**

Spiritual health practitioners bring unique and specific expertise to psychedelic-assisted therapy based on their training and professional experience. They are skilled at interprofessional collaboration in a way that complements other clinical team members. Psychedelic-assisted therapy teams may benefit from including spiritual health practitioners. In order to ensure rigorous standards and quality care, further efforts to delineate the roles and necessary qualifications and training of spiritual health clinicians for psychedelic-assisted therapy are needed.

## Introduction

Psychedelic assisted therapy (PAT) is an experimental treatment with evidence of effectiveness for a range of difficult-to-treat conditions [1] including treatment-resistant depression [2, 3], posttraumatic stress disorder (PTSD) [4], and substance use disorders [5, 6]. PAT involves the administration of a psychedelic compound, (e.g., psilocybin or MDMA), coupled with psychotherapeutic intervention or support [7, 8]. However, there is currently little consensus on *who* should facilitate the therapy, and PAT that includes classic psychedelics [c.f. ketamine assisted psychotherapy [9]is not currently an approved treatment for any condition. Due in part to the long history of psychedelic use in ceremonial or spiritual contexts [10, 13], and because of the prevalence of patients reporting spiritual experiences while undergoing PAT [11–14], some spiritual health practitioners (SHPs) have numbered among the facilitators in PAT, in both legal and extralegal i.e., “underground” settings. The specific roles, functions, and competencies of SHPs in PAT have not been systematically studied or identified, posing an obstacle to the development of guidelines or recommendations for this class of practitioners in PAT. In order to develop evidence-based principles for PAT, the roles of facilitators are important to define, as well as the areas of expertise that are most relevant to supporting patients’ experiences in these treatments. The present study aimed to address this need by examining the experiences of SHPs who have facilitated PAT, with the aim of identifying key areas of competence and activities fulfilled in PAT by this class of professionals.

### Roles of SHPs in PAT

The roles of SHPs in PAT may be especially relevant in light of recent calls for more rigorous protection of patient autonomy and safety in PAT [15], and to improve responsiveness to spiritual content in these therapies [16]. Psychedelic dosing often produces highly impactful, and sometimes challenging, experiences with pronounced effects on spirituality, worldview, and metaphysical perspectives [17]. Much of the emphasis in psychedelic-assisted *therapy* is on ensuring that these experiences lead to beneficial outcomes through the actions of the participant and the care team. Notably, to support beneficial outcomes, PATs typically include a preparation period prior to the dosing session and an integration period afterwards, in order to maximize the therapeutic value and minimize challenges related to the experience. Preparation includes setting an intention, receiving psycho-education, practicing coping skills, and building rapport with the facilitators. Dosing with the psychedelic compound occurs in a safe environment in the presence of facilitators, who offer support or guidance when appropriate. Integration involves assisting the participant in making meaning of their experience, and finding ways to carry beneficial aspects of the therapy forward into their lives. In their roles as facilitators on the care team, SHPs may have unique, and uniquely valuable, contributions to support the participant’s wellbeing and helping them benefit from PAT.

### The roles of spiritual health clinicians in healthcare and their status in PAT

SHPs historically served as chaplains in medical and military settings to provide religiously-based pastoral care to those who share their denomination [18–20]. Although the contemporary profession of chaplaincy is still practiced in medical and military settings, it has come to focus on providing spiritual support for people of any or no religious affiliation. SHPs in clinical settings are predominantly trained in Clinical Pastoral Education CPE through the Association for Clinical Pastoral Education (ACPE) [21]. Qualification as a Board Certified Chaplain involves completion of 1600 clinical and educational hours, 2000 additional hours of clinical practice, endorsement from a religious/spiritual community, submission of four position papers, and committee approval based on demonstration of 31 competencies [22]. SHP Common Competencies designated by the Association for Professional Chaplains (APC), ACPE, and a consortium of spiritual care organizations cover a broad array of spiritual care-related knowledge and skills, including attention to matters of power dynamics, systemic justice concerns, and applied clinical ethics [23]. Furthermore, the training and formation for SHPs are distinct from other clinical disciplines. In addition to the requirements for board certification, training typically includes several years of discernment and formation in seminary and theological training institutions, as well as spiritual direction and direct clinical training during CPE in order to further develop pastoral/spiritual care responses and skills. Endorsement by congregations and ecclesiastic bodies provides a degree of social and community accountability.

A core professional role for SHPs is to address spiritual needs, which are defined as concerns deemed sacred by a person within their life and may or may not include religious components [16, 24]. Given the prevalence and importance of spiritual content in psychedelic experiences, and its potential as a mediator of therapeutic effects [7, 11, 13, 14], SHPs are uniquely positioned to contribute to PAT in ways likely to optimize its therapeutic benefit. Further, although PAT is considered an investigational treatment in contemporary medical settings, there is an extensive history of religious and spiritual practitioner involvement in psychedelic ceremonial use of psychedelics, and more recently in administering psychedelic therapies [10]. Spiritual leaders and healers from Indigenous entheogenic traditions, in particular, have extensive expertise, formation, and training with psychedelic substances, and may practice with these medicines in a variety of contexts. This communally based formation and practice may differ from that of SHPs within a clinical environment, but represents a parallel vector for the inclusion of spiritual professionals in the therapeutic use of psychedelics. Despite the relevance and prevalence of spiritual experiences for PAT study participants and SHPs’ specialized training in providing spiritual care, at present the roles and perspectives of SHPs have been under-represented in the PAT research literature.

In the context of PAT, role definitions or expectations for SHPs as members of the care team have not been an explicit part of treatment protocols, although exceptions exist [25]. Mental health clinicians most commonly serve as therapists within PAT clinical trials. However, training in spiritually informed care is a gap among this group of professionals; a study of 550 US mental health clinicians found that 70% reported receiving no training in religious or spiritual competence [26]. Currently, SHPs are involved in PAT facilitation in medical or research settings primarily when they have dual licensure/certification as a psychotherapist, or when there is a role in the protocol for a facilitator outside of mental health practitioner fields. Settings in which SPHs are able to legally conduct PAT are limited and include Food and Drug Administration (FDA)-approved clinical trials with MDMA and psilocybin, Ketamine-Assisted Therapy, and retreat-based settings outside of the US. However, due to the legal status of psychedelic substances, PAT is not currently practiced frequently in clinical settings.

### The present study

This study conducted interviews with SHPs who had experience facilitating PAT in legal settings. We aimed to identify themes representing how SHPs conceptualized their roles, competencies, activities, and other contributions to this treatment. Understanding SHPs’ perspectives about their role in treatment may yield insights that enhance benefits to patients undergoing PAT and may lead to the development of frameworks for conceptualizing the roles of different members of interprofessional teams in PAT, including SHPs.

## Methods

### Participants

This study recruited SHPs who had experience facilitating PAT, who also had least one of the following: (1) at least one unit of CPE (300 clinical and 100 educational hours), (2) ordination or status as a religious leader, (3) either a Master of Divinity or other post-baccalaureate theological degree, or (4) specific training in spirituality in psychedelic work (i.e., beyond what is offered in PAT certification programs). Recruitment took place from March 20, 2022-June 15, 2022. The study focused on eliciting the perspectives of SHPs who facilitated PAT in settings where it was legal, inclusive of ceremonial/retreat settings and clinical research environments. Because psychedelics are not currently legal under US federal law, in order to minimize legal risks to participants in the event of confidentiality breach this study focused on SHPs facilitating in legal contexts. The inclusion criteria were intentionally broad because this is an emerging field without an established role for SHPs. The study was approved by the Emory University Institutional Review Board and informed written consent was obtained from study participants.

Participants were recruited via emails sent to the *Transforming Chaplaincy Psychedelic Care Network* [16], of which the principal investigator (CP) is a co-convener. Given the formative stage of the present research, this recruitment method was selected in order to facilitate adequate recruitment, although it introduces the possibility of a biased sample of responses due to a shared network between the PI and participants. Snowball sampling was also used, and recipients were encouraged to share the recruitment details with their networks. The SHPs interviewed in this study identified with a variety of terms (e.g., chaplain, facilitator, guide, therapist, spiritual health clinician, spiritual care provider). For the purposes of this manuscript, “SHP” refers to the SHP interviewees in the study. “Participant” is utilized to describe those receiving PAT.

### Measures

SHPs were interviewed via the Zoom telehealth platform, except for one for whom poor internet connectivity required conducting the interview via a written email exchange. A 14-question semi-structured interview guide was used for the interview process (see S1 Appendix). Interview questions covered a variety of topics relevant to the role of SHPs in PAT, including opinions about the importance of personal experience with psychedelics, ethical concerns in PAT, spiritual outcomes of PAT, and questions relating to the emerging field of SHP-engaged PAT. Interviews were recorded and transcribed utilizing the auto-transcription feature of Zoom. All transcripts were manually de-identified and corrected after automated transcription by two researchers, including the interviewer (PI). When the transcripts were unclear, they were checked against live recordings for accuracy.

### Data analysis

Thematic analyses were conducted with the aid of MAXQDA version 22.2.00 qualitative analysis software, relying on both inductive and deductive approaches [27]. Deductive analyses were based on pre-existing research questions formulated by the principal investigator (CP) through her familiarity with the field of chaplaincy and PAT. Questions relevant to this analysis included: In what ways do SHP skills and knowledge translate to PAT? What activities do SHPs engage in through phases of PAT treatment? How do SHPs interface with other disciplines? In what ways do SHP formation, training, and professional expectations impact practice in PAT? Inductively, the material was allowed to inform the emergence of codes and themes, with novel themes generated when interview material was relevant to the central study questions but did not correspond with pre-existing criteria.

First, a subset of transcripts were coded by one investigator (CP), who created a code book. Then a subset of the transcripts were independently coded by a second researcher (EB) using the code book. The two coders then revised code definitions and generated new codes when appropriate. Based on agreed upon changes, investigator (CP) went back to revise previously coded material and completed coding of all transcripts. Based on review of data from the 15 SHPs included in this study and cessation of new code identification, it was deemed that saturation had been achieved.

One of the themes that emerged in the analysis was “Contributions and Perspectives of SHPs with respect to PAT”, which included descriptions of the nature of SHPs’ contributions, their roles, and their insights on PAT. For the present analyses, this theme was further examined for sub-themes. Sub-themes comprised topics that addressed unique and specific areas of focus for SHPs in facilitating PAT. Subthemes were expected to overlap, and so the codes were not developed to be mutually exclusive (i.e., one quote could fit multiple themes). Correspondingly, instead of relying on necessary-and-sufficient definitional characteristics for themes, themes were generated and applied based on prototypical characteristics of the theme rather than boundary conditions.

After a set of sub-themes emerged based on the first author’s initial analyses, these were discussed with the entire authorship team to establish concurrence with the data, relevance to applied topics in PAT, and validity from the interdisciplinary standpoints of practiced spiritual healthcare, medical anthropology, clinical psychology, and psychiatry (JM, RP, BD, GG, DK, RP). This process led to a revision of 6 initial themes to 7.

## Results

### SHP characteristics

Sixteen SHPs consented to participate. One SHP’s interview was excluded when it was determined they did not meet a priori inclusion criteria (their experience with PAT was not within a legal setting). SHPs were from North (n = 14) and South America (n = 1). SHPs had experience practicing in one or more of the following settings: clinical trials (n = 7), private practice (n = 1), virtual PAT (n = 1), clinic (n = 1) and retreat/ceremonial contexts (n = 6). One SHP had experience in both clinical trials and retreat/ceremonial practice. One SHP was also a licensed mental health professional. See Table 1 for participant characteristics, context of practice, and type of medicine.

**Table 1:** Demographics and characteristics of SHPs.

Age*	
Mean	46.57
SD	13.38
Gender	
Female	9 (60%)
Male	6 (40%)
Other	0 (0%)
Race/Ethnicity of SHPs	
Hispanic/Latinx	3 (20%)
White	12 (80%)
Black	0 (0%)
Asian	0 (0%)
Native Hawaiian and Other Pacific Islander	0 (0%)
Context(s) of practice	
clinical trials	7 (46.67%)
private practice	1 (6.67%)
Clinic	1 (6.67%)
retreat/ceremonial	6 (40%)
remote (virtual)	1 (6.67%)
Medicine type(s)	
Ayahuasca	2 (13.33%)
Psilocybin	10 (66.67%)
Ketamine	7 (46.67%)
LSD	1 (6.67%)
MDMA	2 (13.33%)
Cannabis	1 (6.67%)
Kambo	1 (6.67%)

*One participant declined to give their exact age and stated “late 20s”.

### Themes

Thematic analysis of the general theme of “contributions and perspectives of SHPs on their roles in PAT” yielded seven subthemes. These seven subthemes included (1) competency and training to work with spiritual material; (2) awareness of power dynamics; and (3) familiarity with non-ordinary states of consciousness (NOSC), (4) holding space; (5) offer a counterbalance to biomedical perspective; (6) use of generalizable therapeutic repertoire when facilitating PAT; and (7) interdisciplinary collaboration. These seven themes emerged in discussions of all the stages of PAT: preparation, dosing, and integration, as well as in discussion about the general role of SHPs and the overall field of PAT. The seven themes describe both *unique contributions* of SHPs in PAT that derive from their specialized training, experiences, and roles (i.e., themes 1–5), as well as *general contributions* consistent with a common skillset shared among clinicians from different training backgrounds and specializations (i.e., themes 6–7). While the unique contributions illustrate specialized dimensions of care, or specialized execution of existing dimensions of care, that SHPs enact in PAT, generalized contributions reflect SHPs’ operation within a shared framework and common competencies that may be important for all team members to have. Examples and descriptions of the seven themes are presented in Table 2. The next section reports the themes in greater detail.

**Table 2:** Contributions of SHPs in PAT.

Theme	Description	Example
Unique contributions		
1. Competency to work with spiritual material	The capacity to work with spiritual material is discussed as a specific area of competency within the purview of SHPs	*“This work fundamentally touches on spiritual care*. *And that is what chaplains are trained in*. *That is their specialty*.*”*
2. Awareness of power dynamics	SHPs’ perceptions of power dynamics in the care setting and in society more broadly are discussed with relevance to PAT	*“We are trained to be quiet and to listen and to interact alongside rather than to lead*, *control*, *guide*, *change*.*”*
3. Familiarity with NOSC	SHPs discuss their familiarity with NOSC and its relevance to PAT	*“This work touches into those non-ordinary spaces and our role is to stand there and to be that bridge*.*”*
4. Holding space	The creation or sustainment of an interpersonal environment that affords engaging with intimate, sacred, or existentially meaningful experience.	*“So much about my training as a chaplain is just being with*, *witnessing*, *holding*, *supporting without needing to fix or change or anything*… *it’s just really a process of allowing*.
5. SHPs offer a counterbalance to biomedical perspective	SHPs fill role of addressing spiritual content, make space for non-ordinary experience, offer balance to biomedical experience, hold the spiritual container of the experience	*“Chaplains provide a counterbalance to the biomedical approach and also open up the space for some profound and perplexing ideas that may sometimes conflict with some of the underlying assumptions in the biomedical ideology*.*”*
General contributions		
6. Use of generalizable therapeutic repertoire when facilitating PAT	SHPs utilize skills typically offered by other professionals engaged in PAT, such as psychoeducation, safety assessment, life history review, etc.	*“We’re also giving participants tools like breathing practices to help ground*, *…We give mindfulness practices or different meditations so we really try to equip the participants…”*
7. SHPs contribute to interdisciplinary collaboration	SHPs collaborate with other professionals on PAT teams, and often have experience in this type of collaboration from healthcare practice.	“*My chaplain training has been absolutely essential*, *particularly in the hospital context*, *where I was working on a on a team collaborating with clinicians… I really do a lot of communicating with the clinicians…”*

SHP- Spiritual Health Practitioner

PAT- Psychedelic Assisted Therapy

NOSC- Non-Ordinary States of Consciousness

### Unique contributions

This subset of themes includes descriptions of SHPs’ preparation, identity, and activities in PAT that constitute unique contributions that the participants recognized in their work. Some themes, such as Theme 1 (competency to work with spiritual material), derive directly from the specialized background and experience of SHPs. Others, such as Theme 2 (awareness of power dynamics), may be relevant to other professions but are attended with unique relevance by SHPs given the scope of their professional skills and responsibilities. Although SHPs work as members of larger treatment teams that have a shared framework, these unique contributions are perspectives on specialized contributions specific to their professional role on the PAT treatment team.

#### 1. Competency to work with spiritual material

Competency to work with spiritual material was a common theme, identified within 11 SHPs’ interviews. This theme refers to addressing, understanding, and responding to spiritual and religious material, such as beliefs, experiences, and practices relating to God, the transcendent, divine, the universe, and other supernatural or sacred constructs. A key throughline across most SHP interviews was that SHPs are specifically trained to respond to spiritual content and experience, which is a frequent experience of participants undergoing PAT. In this regard, SHPs discussed the value of their personal experience offering spiritual care to patients, and its implications for PAT as a site of spiritual care. This included patients’ positive experiences with spirituality, but also knowledge about how to provide support for participants who had difficult histories with spirituality or had to address spiritual challenges in their current experience.

One SHP reflected that spiritual care is the primary role for a SHP in PAT, both because of their training and because of the experience of PAT for participants:

There is no way around acknowledging that there are spiritual care dimensions to this work that are inherently involved, despite whether somebody comes from a faith tradition or not these experiences bring up matters of the spirit, matters of one’s relationship with life, with death, with all matters of just spiritual dimensions of the human experience.

These dimensions of care may require specialized training and aspects of what is called “formation,” or the development of personal characteristics, as part of that training. “Of course my training helps me. And also just my spiritual perspective in general,” said one SHP. SHPs’ clinical training supports a nuanced understanding and appreciation of spiritual and religious content, which enables them to respond to complex spiritually and religiously oriented experiences. One SHP described working with a patient who experienced a traumatic reaction in relation to a song on the music playlist used during dosing, which brought memories of past abuse in a religious setting. Later in the same session, the participant experienced a connection with a benevolent divine figure that was a part of this same religious tradition. Training in spiritual care and theological reflection allowed the SHP to explore the subtleties of both harmful and supportive religious experiences, while also exploring the participant’s core values and beliefs. This SHP noted that the skills of chaplaincy allowed them to navigate this with subtlety and care:

I think it really helped [to] have a chaplain there to just be able to accept all of the dimensions of that religious experience—all of it was welcomed in and then more integrated… Integrating the generative aspects of religious coping and religious experience with the really abusive harmful stuff and being able to…parse it out…kind of pulling apart the threads and looking at each and then being able to weave this more integrated self through the experience, instead of having these sort of splits…I think that was [a] really positive outcome…if I were to document it [I] might call it ‘religious integration’ or ‘spiritual integration.’

Some SHPs described a capacity to be nimble in response to the variety of religious or spiritual scenarios a participant might encounter in a psychedelic experience, including experiences that reflect different, conflicting, or emphatically non-religious worldviews. In fact, knowledge of spirituality was not regarded by SHPs as only knowledge of a specific theological or religious approach, but a knowledge of the nuances of spiritual care, including non-coercive recognition of atheist and non-religious perspectives. Notably, none of the SHPs described exclusionary or sectarian applications of their knowledge. Many shared a pluralistic framework for conceptualizing their expertise in spiritual care, in which the SHP is prepared and able to respond to any type of theological material with the participant in a manner that is respectful of their belief systems and autonomy.

#### 2. Awareness of power dynamics

SHPs’ awareness of power dynamics refers to SHPs’ attention to interactions that may impose spiritual, religious, or cultural perspectives, which may impinge upon the autonomy of participants, and their awareness of the dynamics of physical touch. It also includes awareness of power dynamics that exist beyond the interpersonal relationship between the participant and clinician, described here as systemic power imbalances and justice-related concerns. These elements were treated as a single theme because they all involve attunement to the power dynamics in the care environment at the different levels where they occur. This theme was present in eleven interviews. SHPs who discussed awareness of power dynamics were cognizant of the many intersecting roles that both the facilitators and the participants in PAT inhabit and referred to the power-related implications of their actions as they fulfilled their roles.

SHPs described an understanding of the role of language in conveying shades of religious connotation and spiritual meaning, which may interfere with a pluralistic support for diverse clients. This enabled attentive and deliberate selection of language, including allowing the patient to introduce their own language around values and beliefs. This care toward language is reflected in a core principle that emerges from spiritual care training: the “living human document.” This is a reference to Anton Boisen’s seminal work regarding hospital-based chaplaincy [28], which highlights the value of honoring the person receiving care, as noted by this SHP:

We seek out what this person is open to, where are they, what do they feel safe with…we are trained to be quiet and to listen and to interact alongside rather than to lead, control, guide, change….the value [of chaplaincy] is of respecting the individual and trying to read the living human document before us.

Non-imposition also involved not intruding upon or invalidating the participants’ emotional experiences, including difficult ones. One SHP said: “when with the family and friends the pain is so evident, and yet we don’t try to change it, we don’t try to numb it, we don’t try to name it, we just hold space and we offer support when we’re acted upon.”

SHPs also described their expertise at conveying their knowledge of power dynamics and allowing it to inform their care, not only during the dosing experience, but during preparation and integration as well. Successfully communicating the SHPs’ role was one way to assure participants that their values, whether religious, spiritual, or not, would be respected. One SHP noted:

My only goal is to help support them in their own meaning making process, whether that’s an atheist, agnostic, spiritual but not religious, religious. What we do in spiritual care is help people deal with the human condition. And I think once you name that and really make it explicit in the preparation stage, people have permission to be and act and interpret however they want. That really empowers them to feel more at ease and then they feel more confident.

Discussion of boundaries around touch in preparation stage was a key element in supporting participant empowerment as discussed by SHPs. Physical touch is commonly used as a support strategy in psychedelic sessions and can risk harm if not approached appropriately [29]. Multiple SHPs talked about the dynamic around physical touch and the importance of employing a strategy during preparation for discussing boundaries and a plan relating to physical touch. Participant autonomy and safety relating to physical touch were priority in several SHP’s interviews.

Cultural humility, a value embedded within spiritual care training and certification programs, was a recurring aspect of SHPs’ attention to power dynamics. Directly stated by one SHP, “we do not impose our de facto belief systems or cultures onto others.” Many references to cultural dynamics were consistent with the principles of cultural humility even when that exact phrase was not used. The values of cultural humility are consistent with attention to the larger context of systemic inequality and historical inequities, including concerns around cultural appropriation of psychedelic medicines. One SHP described chaplains’ contributions of respectfully engaging indigenous traditions while being cognizant of possible cultural appropriation in the context of psychedelic medicine:

I think chaplaincy has a lot to add when it comes to respecting indigenous religious and spiritual traditions that have been using these medicines for millennia. Chaplains can be the champions not only for access but also answering questions about appropriation. That’s where a chaplain’s cultural competency becomes essential. How do we respect the history of these medicines, where they come from, the cultures that they come from? How do we utilize these in a medicinal or spiritual context in a way that is respectful of these things?

#### 3. Familiarity with non-ordinary states of consciousness (NOSC)

SHPs’ awareness of NOSC was discussed by eight SHPs. The topics included within this theme comprise: SHPs’ personal familiarity with NOSC, their awareness of the importance of these states for participants undergoing PAT, and SHPs’ responsiveness to these states. SHPs’ awareness of NOSC was acquired through multiple kinds of experiences, such as care for the dying, and experiences with contemplative, prayerful, and meditative practices. Awareness of and comfort with NOSC was described as a key capacity of SHPs in PAT.

Some SHPs considered familiarity with NOSC to be central to their professional identity and responsibilities. One SHP described chaplaincy as involving “the whole of a person, and innately that includes spirituality in the sense of the meaning and the connections and the relationship that we have to that which is mysterious, transcendent, bigger than ourselves, and deeper than our conscious everyday minds and behaviors.” They saw part of their role as helping to engage with the non-ordinary and building a “bridge” to it, including NOSC.

A variety of experiences beyond PAT were invoked to describe the importance of NOSC in SHPs’ duties and professional experience. Some SHPs described the importance of working with NOSCs while being present with the dying, an important part of their existing professional repertoire that they have been able to learn from and translate to PAT. One SHP described their role in such experiences:

Chaplains have expertise in end-of-life matters when issues of death or experiences of life outside of the present moment, experiences of…we might call them spiritual experiences, peak experiences, religious experiences, experiences of altered consciousness, spiritual visitations, visions.

For multiple SHPs, the appreciation of NOSC as sacred or transcendent was connected to the previous theme of non-imposition. Recognition of the magnitude of such experiences was paired with a hesitancy to foreclose their meanings, and with humility about imposing one’s own lens on such an experience, including within the context of PAT.

SHPs also described how their familiarity with NOSCs helped them to normalize these experiences. Indeed, the SHPs illustrate a kind of complementarity in their accounts of NOSC: on one hand, they describe the importance of treating them with reverence and appreciation. On the other hand, they describe the everyday nature of such experiences, and their willingness to regard such experiences as unique but not unusual. While NOSC may not be ordinary to the participants or to other PAT professionals, they can be ordinary for SHPs without losing their significance or vitality:

…when someone has some sort of weird experience or a deceased loved one they’re grieving comes and visits them in the dosing session, or they need Jesus or an angel. That’s the spiritual care. It’s everything we’re trained for. That’s our bread and butter.

#### 4. Holding space

For this analysis, holding space refers to the creation or sustainment of an interpersonal environment that affords engaging with intimate, sacred, or existentially meaningful experience. This theme included SHPs’ descriptions of being present with the patient, calmly and with supportive, present-moment awareness, and observing internal and external activity with readiness to respond to what may arise [30]. Holding space is also consistent with ritualized and ceremonial practices that many SHPs are trained in through their religious histories and ordination processes. Although holding space shares elements with other clinicians’ professional activities (e.g., empathic listening), SHPs’ emphasis on contemplative and ritual aspects of this activity singles it out as an area of particular emphasis for them. Nine SHPs provided examples of holding space throughout the PAT process. SHPs described the need to respond in a way that was not merely clinical but also spiritual and even ritualistic. One SHP described it this way:

I feel like chaplains are trained to hold the unexplainable, the mystery, the weird, the liminality. So much about my training as a chaplain is just being with, witnessing, holding, supporting without needing to fix or change or anything… it’s just really a process of allowing. I really see us as doulas in a lot of ways—like there are death doulas and birth doulas—but we are almost like psychedelic experience doulas that can just be present with the experience and support without needing to get in there with any concrete tools.

SHPs described holding space in a way that was consistent with the clinical and therapeutic priorities of the treatment. For example, one SHP’s description of holding space included applications to consent, safety, and planning:

… in the preparatory stage a chaplain engages in a ritualized creation of the container, establishing consent, holding the space, and more or less bringing the elements of intentionality, safety, and a solid plan into existence.

Holding space may manifest in different ways across the continuum of the PAT, but often included an attention to the SHPs’ own experience in order to ensure that they were able to provide a supportive presence for the participant. In this way, holding space was distinct from presenting a ‘blank slate’ or compartmentalizing one’s experience; rather, it involved attending to one’s own experience so that the SHP may show up in a particular way. One SHP’s description of holding space during the dosing conveyed a balance of attuning to their own experience, and ensuring that they were able to actively support the participant’s experience without introducing agitation:

If I’m agitated or moving or hungry and not taking care of my body or thirsty or I have to use the restroom, they’re going to be more aware of that than they would normally, or at least experience that I’m not fully engaged with them…it’s a very dynamic following of energy and attendance. What it’s not for me is “interventions” or trying to produce some results… I might ask a few questions or reflect something back for them. But often I’ll track that and maybe in an integration session bring questions or other things, but in the actual medicine session is just allowing the medicine and the participant to do their magic.

It is thus relevant to note that holding space may be conceptualized as distinct from an intervention or therapeutic technique. It is not done in order to achieve any particular outcome, save the participant having an authentic and safe experience with the treatment. Nonetheless, as this SHP described, the capacity to hold space may enable the participant to have experiences that might subsequently become material for more interventional activities during integration.

One SHP who discussed the ceremonial qualities of holding space went on to describe their relevance to emotional difficulties that can arise during PAT:

When there are challenges that come up in the experience a lot of what I…or what my team would be doing would be trying to assist people through those edges. Sometimes that can take the form of just presence or mindful touch or just making sure somebody knows that there’s somebody there. A lot of times what can be really hard and terrifying really is when you’re in a very challenging space and you feel alone, and you don’t feel like anybody’s there.

This statement also highlights a relational quality to holding space. While careful not to impose on their experience, holding space is an attempt to ensure that the participant does not feel that they are alone, and to convey that someone is with them through their experience.

#### 5. Counterbalance to biomedical perspectives

Eight SHPs described ways in which their work as members of the PAT team provides a counterbalance to biomedical perspectives patients generally encounter in clinical care. Correspondingly, this theme refers to SHPs complementing other perspectives on the interdisciplinary care team through addressing spiritual concerns which might not otherwise be addressed, through balancing the biomedical approach when needed, through making space for non-ordinary experience, and through honoring the spiritual container that has been held in indigenous practice with psychedelic medicine.

One SHP described how commonly participants’ psychedelic experience is expressed in spiritual language. These spiritual registers may not be captured by the scientific perspectives or terminology that participants might otherwise encounter in medical settings, which may result in leaving important aspects of their experience unaddressed:

…science is still working to try to understand how these medicines work and how people’s experience is expressed, how the positive outcomes are expressed… in the language of the spiritual domain. People draw on cultural traditions to make sense of their experience. It defies certain kinds of measurement and really requires a more holistic approach in how we support and understand and develop this work.

SHPs did not describe excluding any accepted medical strategies; rather, they described their role as offering the PAT team a capacity to integrate more than biomedical approaches might capture on their own. For example, SHPs described addressing connections with ancestors, experiences of feeling one with the earth or universe, or battling powerful spiritual forces. One SHP reflected:

…the nice thing about working with a chaplain is that we are used to a lot of weird, and that’s okay. Chaplains provide a counterbalance to the biomedical approach and also open up the space for some profound and perplexing ideas that may sometimes conflict with some of the underlying assumptions in the biomedical ideology.

One SHP reflected on the importance of this for the participant, who may have an experience deeply rooted in spiritual or religious experience, as well as for the field, which is developing in its understanding of the breadth of impact of these medicines.

We’re starting to explore psychedelics as kind of a medical intervention, but how do we also recognize the spiritual value of these substances, of these medicines, such that it’s not just a medical intervention for sick people, but it’s a spiritual intervention for well people who are exploring particular spiritual goals. I think having chaplains involved in this work introduces that critical distinction of saying this isn’t only a medicalized treatment. There is something else at work here. There’s something spiritual and emotional going on here as well. And I think just having chaplains involved in the work puts some kind of inherent validity on that approach.

The presence of the SHP acknowledges a multitude of ways in which a participant’s life is connected with the sacred—through their spiritual/religious tradition, their personal history, their core relationships, and the other places they find meaning in their life.

An important aspect of counterbalancing biomedical perspectives included recognizing the utility of skillfully applying indigenous approaches to psychedelic healing. This SHP highlighted the historical practice of psychedelic medicine in indigenous communities and recognized how some of these rich practice histories can be appropriately utilized in clinical settings by SHPs:

Traditionally these medicines…in indigenous communities that work with medicine… are always held in a spiritual context and spirituality is never separate from anything else…And I think we really do a disservice to clients if we don’t hold it in a spiritual context because it’s such an important part of the work.

While valuing indigenous practices was highlighted by several SHPs in this study, as previously mentioned in the theme of *attention to power dynamics*, SHPs also emphasized the importance of avoiding misappropriation.

### General contributions

This subset of themes includes SHPs’ descriptions of their contributions to PAT vis a vis a generalized clinical skillset. Its two included themes, 6 (SHPs make use of a general therapeutic repertoire) and 7 (SHPs contribute to interdisciplinary collaboration), reflect attention to two aspects of clinician roles that were recognized to be an important part of the participants’ work on PAT teams. The identified themes suggest that the qualities of patient-facing clinical roles, as well as the importance of collaboration among staff on multidisciplinary care teams, may be particularly relevant to the duties and responsibilities that participants have experienced in the course of facilitating PAT.

#### 6. SHPs make use of generalizable therapeutic repertoire when facilitating PAT

Eleven of the 15 SHPs described a range of activities in PAT that directly relied on a set of generalized, interpersonal therapeutic skills shared among other clinical professionals. These included: assessment, taking a life history, psychoeducation about the treatment process in PAT, teaching and practicing with coping skills, offering a supportive presence, helping patients make meaning, and helping with integration of life experiences and themes.

The initial application of transdisciplinary therapeutic skills often occurred during participants’ preparation, when SHPs were sometimes involved in initial assessments. Assessments included informal evaluations of participant characteristics and histories that could impact their treatment, as well as formal evaluations for SHPs who are trained to conduct mental health evaluations. One SHP described their role in, as well as their rationale for careful assessments of patients considering PAT:

There would be an assessment of the state of mental health, medications that people might be on or not. If you have an active addiction, this wouldn’t be a person who would be safe to go into this experience. If they’re in acute trauma or in the sense of an unsafe intimate partner, this would not be a good time to move into it. Making sure there’s enough stability and preparedness to enter in and enter out because the work by its very nature is going to become somewhat destabilizing and somewhat dysregulating as we’re moving through transition and we’re moving through trauma and we’re even discovering things that we didn’t necessarily identify before.

Another type of activity that often began during preparation was informing participants about PAT and setting expectations. SHPs remarked on the importance of equipping patients to “navigate” the experience and provide information or techniques that could assist them once they began to feel the psychoactive effects of the treatments during dosing. SHPs mentioned specific skills, “We’re also giving participants tools like breathing practices to help ground…We give mindfulness practices or different meditations, so we really try to equip the participants.” Another SHP described the importance imparting specific information:

Preparation also includes just some education—call it psychoeducation or spiritual education. How do you navigate in the internal world? It’s a bit like teaching people how to go skiing or something… There’s the intention to welcome whatever your mind choreographs and presents to you on the particular day when you take the psychedelic.

This informative approach also involved communicating a stance toward experience and a contemplative sensibility. The SHP continued:

And whatever comes, the intention is to meet it, to welcome it, to embrace it, to explore, not to control it, not to label it…So there’s a certain courage and there’s a decision to trust. It’s not just being passive.

Finally, the educational and informative roles of SHPs comprised setting expectations, including maintaining a realistic sense of what PAT might (and might not) achieve.

I think one of the risks is feeling like psychedelics is a silver bullet or magic something that will cure all, which is obviously not true and [we] try to frame this in a much larger context of equipping participants with tools and a bigger picture.

Like many other health professionals, SHPs might cultivate a therapeutic presence that can be used to support participants throughout the treatment. For the day of the dosing session, SHPs described the importance of offering a “grounding presence” to participants and other staff:

As they arrive, many people have high anxiety coming into a ketamine session, so our role is to be a grounded, clear, calm, compassionate presence.

During the integration portion of the treatment, while continuing to include an emphasis on general therapeutic skills, SHPs also paid additional attention to the broader context of integration, which included families, communities, worldviews, and life narratives. Thus, SHPs blended general therapeutic skills with their unique areas of competence, such as guiding participants through rituals to help integrate.

Our work is not simply to comfort the people we work with, but also to really assist in integration and healing through elements of story, through elements of ritual, and through elements of the heart. [What] I would want healthcare administrators to know is that we chaplains really have a discrete tool belt of interventions that can assist in processes that feed directly into wholeness and healing and wellness.

#### 7. SHPs contribute to interdisciplinary collaboration

Eleven of 15 SHPs described interdisciplinary collaboration on PAT teams. This activity that is common for SHPs is part of a generalized clinical skillset that is shared by many other disciplines, and is also a useful element for PAT. Interdisciplinary collaboration is defined as working with physicians, mental health practitioners, integrative and complementary health professionals, and other clinicians for the benefit of the patient’s experience. Within healthcare environments, SHPs are accustomed to interdisciplinary team work. As noted earlier, it is common for SHPs to round with physicians, social workers, nurses, pharmacists, and other members of the care team. SHPs are often invited to offer their perspective on patient and family concerns from a spiritual health lens. SHPs described the translation of their foundation in collaborative medical care to interdisciplinary PAT teams.

My chaplain training has been absolutely essential, particularly in the hospital context, where I was working on a team collaborating with clinicians… I really do a lot of communicating with the clinicians around kind of particular safety thresholds for the session. But I also take time to write particular […] notes for the clinician after our individual session, so that they really get an idea of how Session One went… I like to fill in the clinician on what the experience is like, how the client is feeling about their treatment process, what the content of the sessions were like, what kind of goals they’re pursuing… Clinicians [in PAT] are a combination of MDs, DOs, psychiatric nurse practitioners, and maybe even some physician assistants.

Interdisciplinary collaboration was also described as a part of retreat/ceremonial contexts, in which six (40%) of the SHPs also had experience. For example, one SHP described group grief retreats for participants whose child has died. These retreats included multidisciplinary clinical teams working together. The SHP emphasized the comprehensive assessments conducted on the retreats, which integrated input and perspectives from various disciplines:

In our team for the grief retreats we have two palliative care physicians who are MDs, one of whom is also a chaplain, which is really lovely. We have a psychiatrist on that team, and I work as a chaplain/therapist. We all consult with one another. Before people ever go on retreat we do a really full comprehensive assessment—physically, emotionally, spiritually, medically for these people, to understand the full complexity of their situation.

SHPs explicitly discussed the positive collaboration and mutual understanding between roles that they experienced on PAT teams, and the factors that contribute to good collaborative relationships. One SHP described the positive nature of their relationship with the other team members. They referred to relationship-building, and recognition of their distinct skillset by other team members, which helped them join together to offer supportive care to their participants:

I would say that I have been able to build a relationship with the doctor and the rest of these licensed therapists. I’m well accepted into the group and they believe that I have something to offer. They acknowledge that the type of pastoral counseling, ethical counseling, moral counseling, the kind of things that chaplains …would interact with is a bit different than the therapy sessions that licensed therapists have. But we all acknowledge that we’re all on the same team we’re all trying to support and equip the patients that we’re working with for healing.

Across multiple examples, SHPs’ also referenced their personal background and experience working on medical teams in order to provide models of successful interprofessional collaboration. For instance, one SHP with experience in palliative care referred to it as an exemplary model for collaborative care.:

I think ideally there’s an interdisciplinary team. Palliative care is a good model. It would be good to have a team that represents all the dimensions of health and well-being–the spiritual, psychological, physical, social dimensions of care. Spirituality should be included in that integrated care plan, and chaplains are specialized in spiritual assessment and meaning making, and should be represented on the team.

Just as their experience in palliative care helped to shape their approach to interdisciplinary care in general, they saw palliative care as a potential model that the emerging field of PAT might draw upon to provide interdisciplinary treatment.

## Discussion

At this critical juncture in the expansion of research and practice in PAT there is a need to identify and optimize effective configurations of clinicians on therapy teams and specific components of optimal PAT. With the potential of near-future approvals by the FDA for MDMA-assisted therapy and psilocybin-assisted therapy for the treatment of specific conditions, PAT is already being taught by a number of organizations, some newly established [31–37]. Although there is growing recognition of the importance of spirituality in PAT [16, 38, 39], and despite SHPs’ long-standing involvement in PAT, their roles as members of PAT care teams are still poorly specified. Some emerging certifying organizations for PAT do not include board certified SHPs among clinicians eligible for PAT certification [16, 38–41]. This study aims to identify unique contributions that SHPs make to PAT and describes their varied roles in PAT delivery and on the interdisciplinary teams involved in PAT.

### Gaps in current practice of PAT

Early research suggests considerable promise for PAT as a treatment for a range of psychological challenges. However, there are important concerns about these therapies that have yet to be resolved. Recent critiques of PAT have raised questions about the power dynamics of PAT: some research indicates that psychedelics may make individuals more suggestible in the short term [42], which means that the autonomy and safety of patients may require additional safeguards. Indeed, patient advocacy efforts have brought complaints about coercion and disregard of boundaries in PAT into the public conversation [15, 43]. Another concern related to the application of power in PAT relates to the histories of colonial violence and oppression toward societies that have historically used psychedelics ceremonially, and which continue to affect the marginalization and inequitable access to PAT, making cultural appropriation an important issue to address in PAT. Further issues relate to a lack of consensus on appropriate components of PAT. Although some have argued that spiritual and religious elements should be left out of such treatments [38], the prevalence and likely mediating role of spiritual experiences in PAT has also led to arguments for systematically addressing spirituality in PAT [16]. SHPs are key to this priority, given their specialized training. A recent systematic review of psychotherapies used during PAT found that 45% of studies did not include a therapeutic protocol; clarifying the roles of different clinicians, including SHPs, in psychotherapy would be an important step toward advancing the evidence base on PAT [44].

### Unique contributions of SHP training to PAT

The major themes identified within this study highlight the important training and formation required for SHPs to provide their essential contributions to the interprofessional PAT team. All of the identified themes in this study are closely tied with existing competencies that are established foci for SHP training. For example, *holding space* requires *external* and *internal awareness*, *competent use of self*, and *capacity for self-reflection*, which are embedded expectations in SHP training and professional practice by ACPE and the APC [21, 22]. Many SHPs are formed in communities in which training in ritual and making space for the sacred (i.e., sacralizing) is central to vocational and professional development. Long and frequent periods of sitting in meditation, prayer, and spiritual worship experiences develop the capacity to hold the SHP’s self and the quality of space that are helpful for those undergoing a psychedelic experience. Additionally, in traditional spiritual care contexts such as hospital and hospice settings, SHPs often provide care for persons in states of deep pain, grief, or altered mental status at end of life. Such experiences of powerful emotions or NOSC may align with other NOSC that occur during PAT, including experiences of “ego death.” Their personal experience with meditation, prayer, and other contemplative practice offers a comfort and familiarity for SHPs in preparing patients for NOSC, witnessing NOSC without judgment and with calm demeanor, and assisting in interpreting during integration after dosing in PAT.

Another area of competency is SHPs’ training in, and honed attention to, matters of power, justice, equity and inclusion, which are often deeply embedded within theological education, formation, and training for SHPs. Because ordained persons hold positions of power in relation to both individuals and communities, preparation to take on these positions includes extensive exploration of power dynamics on interpersonal and systemic levels. Preparation of SHPs involves extensive training in understanding one’s cultural/racial/ethnic background, social location, and major life experiences, and the ways that these factors interplay with persons to whom they offer care. Inclusion of SHPs in a treatment team thus offers another perspective relating to power dynamics that concern participants’ identity characteristics (e.g., religion, gender, race, ethnicity, age), which can be associated with vulnerability for the patient. ACPE and APC include learning outcomes and competencies such as developing “emotional availability,” “cultural humility,” utilizing responsible boundaries, taking into account “multiple elements of cultural and ethnic differences, social conditions, systems, justice and applied clinical ethics issues without imposing one’s own perspectives,” and “use one’s practitioner authority as a spiritual care provider appropriately” [23, 45, 46]. Awareness of these elements for the self and the participant are critical in bringing a sensitivity to power dynamics in the therapeutic process. This awareness and sensitivity can extend to religious and spiritual identity as well. Although practitioners of any discipline may hold a particular religious or spiritual identity, SHPs are specifically trained to understand the nuance of belief in relation to other aspects of the self so that they do not implicitly or explicitly impose their religious or spiritual identity onto the participant. The participant will always have a particular, nuanced, complex set of spiritual, religious, cultural identities that require practitioners who can respond with reverence and respect for the living human document in front of them.

It is notable that SHPs’ descriptions of their roles often departed or even inverted commonplace assumptions about SHP characteristics. While spiritual professionals’ involvement in care for patients may raise concerns about proselytization or imposition, especially when patients are in a vulnerable state, when SHPs discussed these issues they primarily emphasized the importance of guarding against transgressions or impositions. The SHPs in this study rather emphasized the balancing of multiple epistemic perspectives, including the religious, spiritual, materialistic, biomedical, indigenous, settler, and traditional. Given that individuals who cope with personally impactful events—especially those that are medically and the spiritually relevant—may seek a range of religious and scientific interpretive frameworks for their experience [47], SHPs may be positioned as allies in this formative search.

### Natural allies: SHPs as collaborative members of PAT teams and future directions

An interdisciplinary approach, such as ones already employed in palliative care teams [48] may offer a uniquely effective care model for PAT. Based on our findings, a dyad of a licensed mental health clinician paired with a SHP is an existing and appreciated arrangement for the delivery of PAT [25] with a history of implementation. This dyad should be collaborative and model trust, openness, and connection for the participant, with both providers engaging in self-reflection and ongoing self-care, and both bringing their specialization. This allows the mental health clinician to provide expertise in therapeutic processes and techniques, mental health assessment and treatment, safety risk and management, and the SPH to provide therapeutic expertise in holding space, spiritual assessment/intervention, responding to spiritual/religious/NOSC experience, and supporting sensitivity to power dynamics. There is some overlap in skills between the two members of the dyad; a well-functioning team will capitalize on this overlap while drawing upon one another’s unique competencies the ultimate benefit of the participant.

In research settings, SHPs may extend their role in balancing biomedical perspectives to the ways in which studies are designed and interpreted. Although biopsychosocial-spiritual models [49] are increasingly valued in medicine, SHPs have considerable expertise in the “spiritual” component of such a model. They may help to recognize the role and impact of spiritual dimensions of PAT treatments because their training prepares them to notice it. As members of research teams, SHPs, may also collaboratively assist with development of study protocols and team development, contributing their expertise to identifying systemic matters of power and justice. For example, SHPs might raise important questions during study design and implementation as elements of cultural appropriation emerge. SHPs may also act as liaisons with indigenous spiritual practitioners in order to enhance clinical practices for the benefit of participants while honoring the historic and ongoing relationships with plant medicines in indigenous communities. Indeed, SHPs can be an important staff resource for developing reciprocity with indigenous rights-holders, particularly when working within established financially stable institutions. As SHPs collaborate with other professionals in study design, they may have contributions for supporting equity, such as how a team may actively recruit persons from marginalized communities to receive the potential benefits of engagement in psychedelic clinical trials by including spirituality and religion in efforts to work with participants’ communities. They may also help to prioritize marginalized communities’ concerns in relation to clinical research.

It is our recommendation that training programs and developing certifying bodies take seriously the contributions of SHPs to PAT and include them in their programs, and that SHPs be considered among those trained professionals who are able to receive training toward future certification, as has been proposed for physicians, advanced medical practice providers, licensed mental health counselors, and social workers. Furthermore, as standards of care are developed, it will be important to not only include SHPs as professionals who can provide PAT, but to also ensure that other professionals are not neglecting the spiritual, existential, religious, and theological concerns that arise in the course of treatment. Given the potential for FDA approval of some psychedelic substances in near future [50], these are important and timely steps for the field.

## Limitations of study

This study should be interpreted in light of several key limitations. The qualitative, retrospective nature of this research is designed to provide rich accounts of SHPs’ experiences and provide a thorough account of their contributions of PAT. However, it cannot address questions of causality or speak to the effectiveness of the therapeutic approaches used by the SHPs. Given the formative nature of this research and to support adequate sampling, recruitment included outreach to participants from a professional network co-convened by the PI. This may have biased inclusion of professionals who share views similar to those held by the PI, and may have biased responses toward those aligned with those views. Further, the sampling of this study was limited in several ways, which may restrict the generalizability of our findings. The study only included persons practicing in legal contexts, which may have excluded perspectives of SHPs with experience in community based (i.e., “underground”) contexts. In community-based settings, SHPs or spiritual guides have been providing psychedelic-assisted care for decades, sometimes building from ancient practices in indigenous communities, suggesting a wealth of knowledge that may inform the conduct of PAT in medical settings. In the field at large, there has been limited research addressing the experience of Black, Indigenous and people of color with PAT [51, 52]. This limitation characterizes the present research about SHPs providing PAT. In our study SHP participants were predominantly white, and the study included limited perspectives of those who identified as Black, Indigenous, and people of color. The SHPs in this study have had extensive experience with PAT, and the data in this study may therefore be susceptible to another kind of selection bias: it is unclear to what extent SHPs who have *not* previously expressed interest in PAT would endorse similar positions, and this question requires further study. Finally, SHPs in this study were exclusively from the Western hemisphere; PAT practice may look differently under different cultural and social conditions, for example in medical systems where chaplaincy does not have an established history. Unique approaches to addressing the concerns expressed by SHPs in this study may be relevant in those settings. For these reasons, data from this research should be interpreted as a first step in understanding the roles of SHPs in PAT. Future work should explore the experiences of individuals from more diverse networks, and those working in underground and in traditional religious/spiritual settings to see whether there may be additional insights beyond those presented here.

Although SHPs discussed the feasibility of working as members of interprofessional collaborative teams from a care delivery perspective, it was beyond the scope of the present study to examine the financial requirements to support that kind of care. Two SHPs in the study identified that SHPs are a cost-effective resource for PAT teams, considering the salary norms of SHPs as compared to physicians, psychologists, and other mental health practitioners. Given recent interest in ensuring that high-quality PAT is scalable and affordable, especially for disadvantaged groups [53], this topic may be a valuable consideration for future studies. The themes yielded in this study were not subjected to member checking due to restrictions in study timeline. Subsequent research with a larger sample of SHPs will be instructive for confirming or refining these themes based on SHPs’ perspectives on their own roles.

## Conclusion

SHPs have had a growing and changing role within many Western healthcare institutions for nearly a century [20, 24, 28, 54–56]. Working alongside physicians, nurses, and other allied professions, SHPs have emerged as essential members of the interdisciplinary care team offering bedside care to patients and their care partners. SHPs use unique skills, knowledge, and activities that may be uniquely useful for PAT. As physicians are competent to respond to patients’ biomedical concerns, and mental health practitioners are competent to respond to psychological concerns, SHPs are competent to respond to spiritual concerns of the psychedelic experience. Persons who are suffering will increasingly seek PAT for relief, and treatment teams will have the opportunity to respond to these needs in a holistic manner, taking into account the full dimension of human experience, inclusive of spiritual dynamics through inclusion of SHPs on their interdisciplinary teams.

## Supporting information

S1 Appendix (DOCX)

## References

[1] Wheeler SW, Dyer NL. A systematic review of psychedelic-assisted psychotherapy for mental health: An evaluation of the current wave of research and suggestions for the future. Psychology of Consciousness: Theory, Research, and Practice. 7[3]:279–315.

[2] Carhart-Harris RL, Bolstridge M, Day CMJ, Rucker J, Watts R, Erritzoe DE, et al. Psilocybin with psychological support for treatment-resistant depression: six-month follow-up. Psychopharmacology [Berl]. 2018;235[2]:399–408. doi: 10.1007/s00213-017-4771-x 29119217 PMC5813086

[3] Goodwin GM, Aaronson ST, Alvarez O, Atli M, Bennett JC, Croal M, et al. Single-dose psilocybin for a treatment-resistant episode of major depression: Impact on patient-reported depression severity, anxiety, function, and quality of life. J Affect Disord. 2023;327:120–7. doi: 10.1016/j.jad.2023.01.108 36740140

[4] Mitchell JM, Bogenschutz M, Lilienstein A, Harrison C, Kleiman S, Parker-Guilbert K, et al. MDMA-assisted therapy for severe PTSD: a randomized, double-blind, placebo-controlled phase 3 study. Nat Med. 2021;27[6]:1025–33. doi: 10.1038/s41591-021-01336-3 33972795 PMC8205851

[5] Bogenschutz MP, Ross S, Bhatt S, Baron T, Forcehimes AA, Laska E, et al. Percentage of Heavy Drinking Days Following Psilocybin-Assisted Psychotherapy vs Placebo in the Treatment of Adult Patients With Alcohol Use Disorder: A Randomized Clinical Trial. JAMA Psychiatry. 2022;79[10]:953–62. doi: 10.1001/jamapsychiatry.2022.2096 36001306 PMC9403854

[6] dos Santos RG OF, Crippa JAS, Riba J, Zuardi AW, Hallak JEC. Antidepressive, anxiolytic, and antiaddictive effects of ayahuasca, psilocybin and lysergic acid diethylamide (LSD): a systematic review of clinical trials published in the last 25 years. Therapeutic Advances in Psychopharmacology. 2016;6[3]:193–213. doi: 10.1177/2045125316638008 27354908 PMC4910400

[7] Cosimano MP. The Role of the Guide in Psychedelic‑Assisted Treatment. Handbook of Medical Hallucinogens. 2021.

[8] Johnson M, Richards W, Griffiths R. Human hallucinogen research: guidelines for safety. J Psychopharmacol. 2008;22[6]:603–20. doi: 10.1177/0269881108093587 18593734 PMC3056407

[9] Drozdz SJ, Goel A, McGarr MW, Katz J, Ritvo P, Mattina GF, et al. Ketamine Assisted Psychotherapy: A Systematic Narrative Review of the Literature. J Pain Res. 2022;15:1691–706. doi: 10.2147/JPR.S360733 35734507 PMC9207256

[10] Winkelman MJ. The Evolved Psychology of Psychedelic Set and Setting: Inferences Regarding the Roles of Shamanism and Entheogenic Ecopsychology. Front Pharmacol. 2021;12:619890. doi: 10.3389/fphar.2021.619890 33732156 PMC7959790

[11] Cole-Turner R. Psychedelic Mystical Experience: A New Agenda for Theology. Religions. 2022;13[5]:385.

[12] MacLean KA, Leoutsakos J-M, Griffiths R. Factor Analysis of the Mystical Experience Questionnaire: A Study of Experiences Occasioned by the Hallucinogen Psilocybin. Journal for the scientific study of religion. 2012;51[4]:721–37. doi: 10.1111/j.1468-5906.2012.01685.x 23316089 PMC3539773

[13] Pahnke WN. The Psychedelic Mystical Experience in the Human Encounter with Death. The Harvard Theological Review. 1969;62[1]:1–21.

[14] Pahnke WN, Richards WA. Implications of LSD and Experimental Mysticism. Journal of Religion and Health. 1966;5[3]:175–208. doi: 10.1007/BF01532646 24424798

[15] McNamee S, Devenot N, Buisson M. Studying Harms Is Key to Improving Psychedelic-Assisted Therapy—Participants Call for Changes to Research Landscape. JAMA Psychiatry. 2023;80[5]:411–2. doi: 10.1001/jamapsychiatry.2023.0099 36988924

[16] Palitsky R, Kaplan DM, Peacock C, Zarrabi AJ, Maples-Keller JL, Grant GH, et al. Importance of Integrating Spiritual, Existential, Religious, and Theological Components in Psychedelic-Assisted Therapies. JAMA Psychiatry. 2023;80[7]:743–9. doi: 10.1001/jamapsychiatry.2023.1554 37256584

[17] Timmermann C, Kettner H, Letheby C, Roseman L, Rosas FE, Carhart-Harris RL. Psychedelics alter metaphysical beliefs. Scientific Reports. 2021;11[1]:22166. doi: 10.1038/s41598-021-01209-2 34815421 PMC8611059

[18] Cadge W, Stroud IE, Palmer PK, Fitchett G, Haythorn T, Clevenger C. Training Chaplains and Spiritual Caregivers: The Emergence and Growth of Chaplaincy Programs in Theological Education. Pastoral psychology. 2020;69[3]:187–208.

[19] Ford T, Tartaglia A. The development, status, and future of healthcare chaplaincy. South Med J. 2006;99[6]:675–9. doi: 10.1097/01.smj.0000220893.37354.1e 16800440

[20] Loewy RS, Loewy EH. Healthcare and the hospital chaplain. MedGenMed. 2007;9[1]:53. 17435653 PMC1924976

[21] ACPE: The Association for Clinical Pastoral Education [https://acpe.edu. 10.1177/002234098804200303 10290151

[22] Association of Professional Chaplains [https://www.professionalchaplains.org/.

[23] APC, ACPE, CASC/ACSS, NACC, NAJC. Common Qualifications and Competencies of Professional Chaplains. Association for Professional Chaplains; 2016 11/07/2004.

[24] Cadge W, Fitchett G, Haythorn T, Palmer PK, Rambo S, Clevenger C, et al. Training Healthcare Chaplains: Yesterday, Today and Tomorrow. J Pastoral Care Counsel. 2019;73[4]:211–21. doi: 10.1177/1542305019875819 31829123

[25] National Institute of Health Clinical Trials. Psilocybin Combined With Multidisciplinary Palliative Care in Demoralized Cancer Survivors With Chronic Pain. 2022.

[26] Oxhandler HK, Polson EC, Achenbaum WA. The Religiosity and Spiritual Beliefs and Practices of Clinical Social Workers: A National Survey. Soc Work. 2018;63[1]:47–56. doi: 10.1093/sw/swx055 29136245

[27] Layder D. Sociological practice: Linking theory and social research London. University of Leicester, UK: Sage Publishing; 1998.

[28] Boisen A. Out of the Depths. New York: Harper and Brothers; 1960.

[29] Brennan WJ, Margo A; MacLean, Katherine; Ponterotto, Joseph G.;. A qualitative exploration of relational ethical challenges and practices in psychedelic healing. Journal of Humanistic Psychology. 2021; 2216782110571.

[30] Wright Glen A. Holding Space: On Loving, Dying, and Letting Go. Berekely, CA: Parallax Press; 2017.

[31] MAPS: The Multidisciplinary Association for Psychedelic Science [https://maps.org/.

[32] Naropa Psychedelic Assisted Therapies Certificate Program [https://www.naropa.edu/academics/extended-campus/psychedelic-assisted-therapies-certificate/.

[33] Compass Pathways [https://compasspathways.com/.

[34] Center For Psychedelic Studies and Research, California Institute for Integral Studies [https://www.ciis.edu/research-centers/center-for-psychedelic-therapies-and-research.

[35] University of California Berkeley Center for the Science of Psychedelics [https://psychedelics.berkeley.edu/training/.

[36] Synthesis Institute [https://www.synthesisinstitute.com/psychedelic-practitioner-core-training.

[37] Rosenbaum D, Boyle AB, Rosenblum AM, Ziai S, Chasen MR. Psychedelics for Psychological and Existential Distress in Palliative and Cancer Care. Current Oncology. 2019;26[4]:225–6. doi: 10.3747/co.26.5009 31548800 PMC6726261

[38] Psychedelic medicine is going mainstream. Who will benefit? The Washington Post.

[39] Ko K, Knight G, Rucker JJ, Cleare AJ. Psychedelics, Mystical Experience, and Therapeutic Efficacy: A Systematic Review. Front Psychiatry. 2022;13:917199. doi: 10.3389/fpsyt.2022.917199 35923458 PMC9340494

[40] Psychedelic Medicine Association [https://psychedelicmedicineassociation.org/.

[41] American Psychedelic Practitioners Association [https://www.thepsychedelicassociation.org/.

[42] Carhart-Harris RL, Kaelen M, Whalley MG, Bolstridge M, Feilding A, Nutt DJ. LSD enhances suggestibility in healthy volunteers. Psychopharmacology (Berl). 2015;232[4]:785–94. doi: 10.1007/s00213-014-3714-z 25242255

[43] Barber GS, Dike CC. Ethical and Practical Considerations for the Use of Psychedelics in Psychiatry. Psychiatr Services. 2023:838–846. doi: 10.1176/appi.ps.20220525 36987705

[44] Cavarra M, Falzone A, Ramaekers JG, Kuypers KPC, Mento C. Psychedelic-Assisted Psychotherapy—A Systematic Review of Associated Psychological Interventions. Frontiers In Psychology. 2022;12. doi: 10.3389/fpsyg.2022.887255 35756295 PMC9226617

[45] Idler EL, Binney Z, Grant GH, Perkins MM, Quest T. Practical Matters and Ultimate Concerns, “Doing,” and “Being”: A Diary Study of the Chaplain’s Role in the Care of the Seriously Ill in an Urban Acute Care Hospital Journal for the Scientific Study of Religion. 2015;49[2]:722–38.

[46] ACPE. Objectives and Outcomes 2016.

[47] Palitsky R, Cooper D, Lindahl J, Britton W. Relationships between Religious and Scientific Worldviews in the Narratives of Western Buddhists Reporting Meditation-Related Challenges. Journal of Contemplative Studies. 2023:1–28.

[48] Fernando G, Hughes S. Team approaches in palliative care: a review of the literature. Int J Palliat Nurs. 2019;25[9]:444–51. doi: 10.12968/ijpn.2019.25.9.444 31585054

[49] Sulmasy DP. A biopsychosocial-spiritual model for the care of patients at the end of life. Gerontologist. 2002;42 Spec No 3:24–33. doi: 10.1093/geront/42.suppl_3.24 12415130

[50] Psychedelic drugs: considerations for clinical investigations. Silver Spring, MD: Food and Drug Administration, June 2023. (https://www.fda.gov/media/169694/ download.

[51] Michaels TI, Purdon J, Collins A, Williams MT. Inclusion of people of color in psychedelic-assisted psychotherapy: a review of the literature. BMC Psychiatry. 2018;18[1]:245. doi: 10.1186/s12888-018-1824-6 30064392 PMC6069717

[52] Fogg C, Michaels TI, de la Salle S, Jahn ZW, Williams MT. Ethnoracial health disparities and the ethnopsychopharmacology of psychedelic-assisted psychotherapies. Exp Clin Psychopharmacol. 2021;29[5]:539–54. doi: 10.1037/pha0000490 34096755

[53] Williams MTL, Beatriz C. Diversity, equity, and access in psychedelic medicine. Journal of Psychedelic Studies. 2020;4[1]:1–3.

[54] Cadge W, Sigalow E. Negotiating Religious Differences: The Strategies of Interfaith Chaplains in Healthcare. Journal for the scientific study of religion. 2013;52[1]:146–58.

[55] Dicks R. Standards for the work of the chaplain in a general hospital. American Protestant Hospital Association Bulletin. 1940;4:1–4.

[56] Jeuland J, Fitchett G, Schulman-Green D, Kapo J. Chaplains Working in Palliative Care: Who They Are and What They Do. Journal of Palliative Medicine. 2017;20[5]:502–8. doi: 10.1089/jpm.2016.0308 28146647

