# Supplementary material for: Spiritual health practitioners’ contributions to psychedelic assisted therapy: A qualitative analysis
Source: PLoS One. 2024 Jan 2;19(1):e0296071. doi: 10.1371/journal.pone.0296071 (PMC10760908; doi:10.1371/journal.pone.0296071)
Supplement: S1 Appendix — (DOCX) [file pone.0296071.s001.docx]

Appendix A

**Interview Questions**

*Can you please describe the context(s) in which you provide psychedelic assisted therapy?*

*What led you into the work of psychedelic assisted therapy?*

*What, if anything, gives you meaning or fulfillment in this work?*

*Do you think it’s important for a chaplain/spiritual health practitioner to have experience with psychedelics, mystical, or non-ordinary states of consciousness to be an effective guide?*

*Can you please describe in as much detail your activity during preparation, dosing sessions and any integration sessions?*

*What would you describe as the beneficial spiritual outcomes or PAT? Do you feel there are any negative spiritual outcomes for people participating in PAT? If so, what are they?*

*How have you interfaced with other practitioners in PAT and if so, can you please describe (e.g., physicians, psychologists, etc.)?*

*How can religious/spiritual guides in communities that utilize psychedelics ritually/ceremonially inform the role of chaplains/spiritual health practitioners in a medical, mental health, or wellness context?*

*What do you wish healthcare administrators knew about the role of chaplains/spiritual health practitioner in this work?*

*Do you play a role with music during psychedelic treatment sessions? If so, what do you do?*

*What role can chaplains/spiritual health practitioners play in tending to ethical concerns in the field?*

*What do you think the emerging field of chaplain/spiritual health practitioner engaged PAT needs at this moment in time?*
